# Robot-assisted left upper lobectomy with pulmonary artery lateral stapled resection after neoadjuvant therapy

**DOI:** 10.1016/j.xjtc.2026.102375

**Published:** 2026-03-25

**Authors:** Lucas Leleu, Benjamin Esnault, Benjamin Bottet, Jean-Marc Baste

**Affiliations:** Department of Cardiac and Thoracic Surgery, CHU Rouen, Rouen, France

**Figure:**
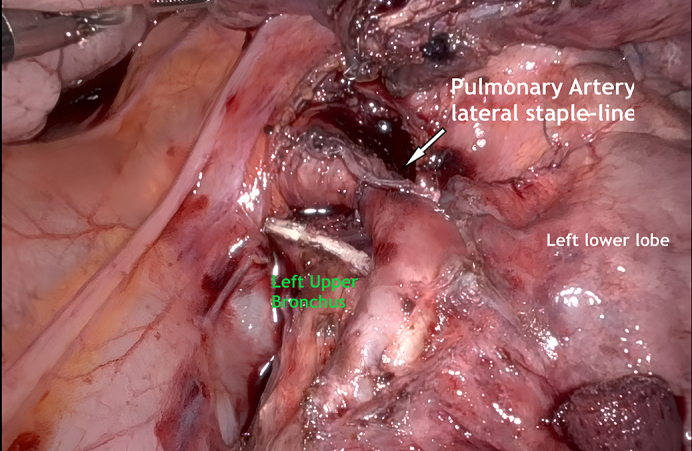
Pulmonary hilum after left upper lobectomy and pulmonary artery lateral stapled resection.

**Central Message**

Fibrotic adhesions may preclude dissection of segmental arterial branches. Lateral stapled resection of the pulmonary artery allows completion of a robot-assisted lobectomy while preserving lower lobe perfusion.

A 61-year-old female patient with a history of smoking was diagnosed with a left upper lobe adenocarcinoma (programmed death-ligand 1 20%). Computed tomography (CT) scan of the chest revealed a 15-mm pulmonary nodule with positron emission tomography–positive hilar lymph node. Clinical stage was cT2N1M0 (TNM 9).

After a multidisciplinary tumor board discussion, the patient received 3 cycles of neoadjuvant chemoimmunotherapy (carboplatin-pemetrexed-nivolumab)^1^ with good metabolic response and was scheduled for a robot-assisted left upper lobectomy with radical lymphadenectomy. Institutional review board approval was not needed. Informed written consent was obtained from the patient for the publication, reproduction, and broadcast of audio-visual and textual material.

Surgery was performed using the standardized French lobectomy technique^2^ (Video 1) in the right lateral decubitus. After initial adhesiolysis and pulmonary ligament dissection (zone 1), a counterclockwise dissection of hilum was performed according to the technique.^2^ Systematic lymphadenectomy exposed the posterior pulmonary artery (zone 2) and aortopulmonary window (zone 3). A dense fibrosis surrounded the upper segmental artery, as commonly observed after induction therapy.^3^

The fissure was opened (zone 5) using the “Colibri” technique, allowing both visceral pleural to slide past each other. Small arterial branches were controlled using energy, leaving a short stump for potential compression hemostasis.

After anterior hilar exposure (zone 4), the left superior pulmonary vein was stapled. The left pulmonary artery was secured with a tourniquet to ensure safe continuation.

The left upper lobar bronchus was stapled. Dissection of the segmental arterial branches was deemed unsafe because of the dense fibrosis. Several factors were considered when evaluating the potential involvement of the pulmonary artery, including the absence of arterial invasion on the initial CT scan, the peripheral localization of the primary tumor, the metabolic response after induction therapy (>80% standardized uptake value reduction), which has been associated with a greater likelihood of major or complete pathologic response in recent exploratory fluorodeoxyglucose positron emission tomography studies,^4^ and the intraoperative periarterial dissection with the presence of a macroscopically safe area for stapling. Therefore, a lateral stapled pulmonary artery resection was performed using two 8-mm vascular staplers.^5^

Indocyanine green confirmed preserved left lower lobe perfusion. Postoperative contrast-enhanced CT scan and 3-dimensional reconstruction (Figure 1) demonstrated a 50% pulmonary artery stenosis with preserved lower-lobe perfusion. The postoperative course was uneventful, and the patient was discharged at day 5. Pathology confirmed an R0 resection with complete pathologic response. CT scan at 1-month postoperatively confirmed maintained perfusion.

**Figure 1:**
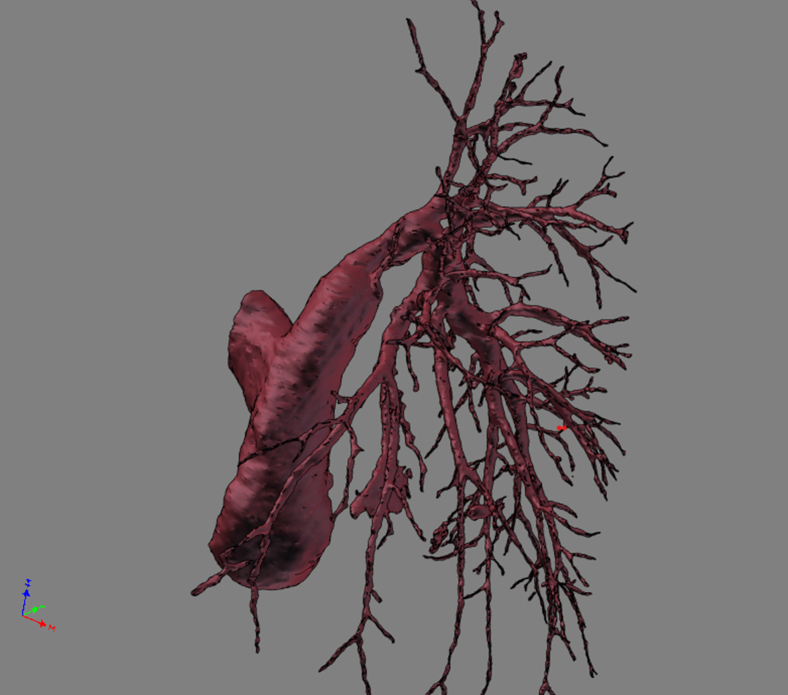
Three-dimensional postoperative reconstruction showing the lateral staple line with preserved arterial caliber.

Compared with formal angioplasty, lateral stapled resection may simplify the procedure and reduce operative time and bleeding risk. The use of 8-mm vascular staplers facilitates passage within the confined hilar space and may limit arterial stenosis. Distal perfusion should be assessed intraoperatively with indocyanine green and confirmed postoperatively by imaging. If arterial invasion is suspected or margins are uncertain, a frozen section analysis should be routinely performed to ensure the absence of microscopic invasion. If margin status remains uncertain, conventional sharp division with formal arterial reconstruction should be preferred.

## Conclusions

In selected postinduction cases, lateral stapled pulmonary artery resection allows safe completion of robotic lobectomy with preserved distal perfusion.

## Conflict of Interest Statement

Dr Baste Jean-Marc is a Proctor for Intuitive. All other authors reported no conflicts of interest.

The *Journal* policy requires editors and reviewers to disclose conflicts of interest and to decline handling or reviewing manuscripts for which they may have a conflict of interest. The editors and reviewers of this article have no conflicts of interest.

## Supplementary material

Video 1 Robot-assisted left upper lobectomy with pulmonary artery lateral stapled resection after neoadjuvant therapy. Video available at: https://www.jtcvs.org/article/S2666-2507(26)00182-3/fulltext.

## References

[1] Forde P.M., Spicer J.D., Provencio M. (2025). Overall survival with neoadjuvant nivolumab plus chemotherapy in lung cancer. N Engl J Med.

[2] Mordojovich G., Hugen N., Bottet B. (2025). New standardized five-zone lobectomy with structured assessment in robotic surgery: the French lobectomy. J Thorac Dis.

[3] Trabalza Marinucci B., Mancini M., Siciliani A. (2025). Surgical techniques for non-small-cell lung cancer after neoadjuvant chemo-immunotherapy: state of art and review of the literature. Cancers (Basel).

[4] Seitlinger J., Salko O., Bulgarelli Maqueda L. (Published online October 28, 2025). Neoadjuvant chemotherapy-immunotherapy in non-small cell lung cancer: can positron emission tomography scan predict complete pathologic response?. Ann Thorac Surg.

[5] Vannucci J., Matricardi A., Potenza R., Ragusa M., Puma F., Cagini L. (2018). Lobectomy with angioplasty: which is the best technique for pulmonary artery reconstruction?. J Thorac Dis.

