# Supplementary figures and images for: Robot-assisted left upper lobectomy with pulmonary artery lateral stapled resection after neoadjuvant therapy
Source: JTCVS Tech. 2026 Mar 25;37:102375. doi: 10.1016/j.xjtc.2026.102375 (PMC13261265; doi:10.1016/j.xjtc.2026.102375)

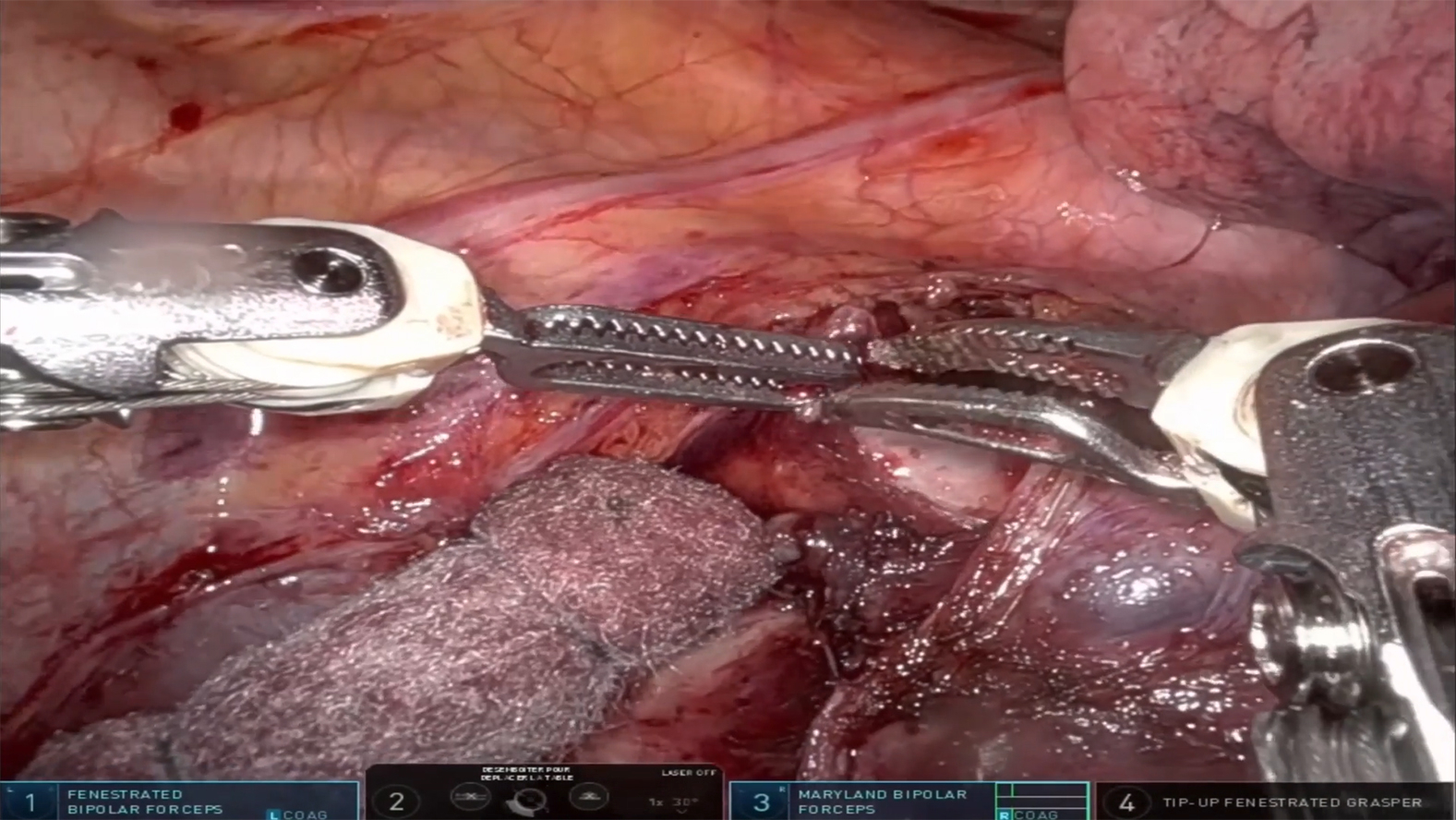

Supplement: Video 1 — Robot-assisted left upper lobectomy with pulmonary artery lateral stapled resection after neoadjuvant therapy. Video available at: https://www.jtcvs.org/article/S2666-2507(26)00182-3/fulltext. [file fx2.jpg]
